# Modeling seed germination data to meet biodiversity conservation needs in the Mediterranean

**DOI:** 10.1111/cobi.70329

**Published:** 2026-05-18

**Authors:** Diana María Cruz‐Tejada, Efisio Mattana, Angelino Carta

**Affiliations:** ^1^ Botany Unit, Department of Biology University of Pisa Pisa Italy; ^2^ Royal Botanic Gardens, Kew, Wakehurst West Sussex UK; ^3^ CIRSEC—Centre for Climate Change Impact University of Pisa Pisa Italy

**Keywords:** conservation, data quality, germination, restoration, seed traits, thermal modeling, Características de las semillas, calidad de los datos, conservación, germinación, modelación térmica, restauración, 保护, 数据质量, 萌发, 恢复, 种子性状, 热响应建模

## Abstract

Seed‐based conservation and restoration programs rely on sound knowledge in fundamental seed biology. However, the adequacy of current data on seed germination to meet biodiversity conservation needs remains largely unexplored. We assessed germination data availability and quality for 1495 Mediterranean species of conservation and restoration interest to determine how much germination information is currently available; whether existing data meet the requirements for thermal germination risk assessment; and which species or ecological features help explain current data gaps. To this end, we used phylogenetic logistic regressions and assessed the adequacy of the available data by modeling the species’ seed germination thermal responses. Germination data were available for 31% of species but adequate for thermal modeling for only 9% of them. Species with data were mainly ecologically widely distributed, produced physiologically dormant seeds, including species suitable for restoration, and had traditional uses. Thermal risk was not phylogenetically clustered, but it was linked to habitat, with species from forests and scrubland exhibiting higher thermal risk. Based on our results, we devised a framework to support conservation and restoration efforts that includes steps to improve data availability (e.g., data gap analysis) and to classify species according to their vulnerability to climate warming. These steps will help distinguish between species that are potentially suitable for restoration programs and those that should be prioritized for conservation and demonstrate how germination information can be modeled and translated into thermal risk assessments to support seed‐based conservation and restoration programs.

## Graphical abstract

Workflow to assess the availability and quality of germination data and assign species' thermal risk in the Mediterranean. (a) Assessment of data availability, including the phylogenetic distribution of the number of records (i.e. available germination data); (b) data quality assessment; and (c) assignment of climate risk categories for species that pass quality criteria. The number and percentages of species with germination data available, extracted from germination data sources for the pool of characteristic species of Mediterranean habitats are shown in (a). The phylogenetic tree represents a wide phylogenetic dispersion, including the most representative clades. Numbers in (b) represent the number of species that passed the modeling criteria in turquoise squares, and the number of species that did not pass the criteria in turquoise circles, as indicated; NTestTemp, Model concavity, Tail drop (not flat), correspond to the modeling criteria applied to the data quality assessment. In (c) is visualized how the thermal risk is calculated, starting from the quadratic modeling representing the thermal seed germination and the assignment of the different risk categories.

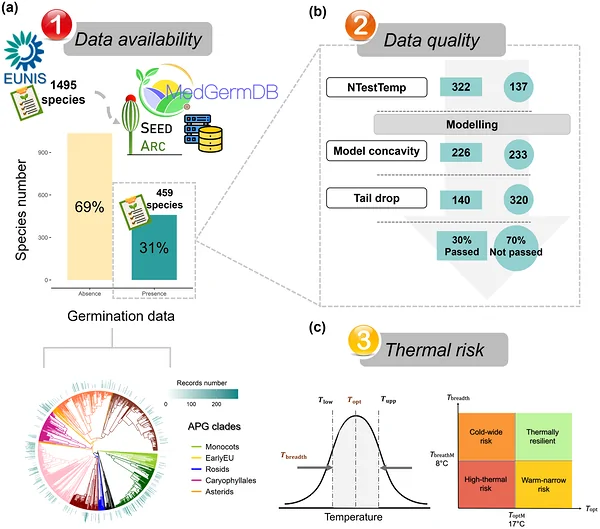

## Abstract (es)

Modelación de datos sobre la germinación de semillas para satisfacer las necesidades de conservación de la biodiversidad en el Mediterráneo

**Resumen:**

Los programas de conservación y restauración basados en semillas se basan en un conocimiento sólido de la biología fundamental de las semillas. Sin embargo, la idoneidad de los datos actuales sobre la germinación de semillas para satisfacer las necesidades de conservación de la biodiversidad sigue sin haberse explorado en gran medida. Hemos evaluado la disponibilidad y la calidad de los datos de germinación de 1495 especies mediterráneas de interés para la conservación y la restauración con el fin de determinar cuánta información sobre germinación hay disponible actualmente; si los datos existentes cumplen los requisitos para la evaluación del riesgo térmico de germinación; y qué especies o características ecológicas ayudan a explicar las lagunas actuales en los datos. Para ello, utilizamos regresiones logísticas filogenéticas y evaluamos la idoneidad de los datos disponibles mediante la modelación de las respuestas térmicas de la germinación de las semillas de las especies. Se disponía de datos de germinación para el 31 % de las especies, pero solo el 9 % de ellas eran adecuados para la modelación térmica. Todas las especies presentaban, en su mayoría, una amplia distribución ecológica, producían semillas fisiológicamente latentes —incluidas especies aptas para la restauración— y tenían usos tradicionales. El riesgo térmico no se agrupó filogenéticamente, pero sí se relacionó con el hábitat, ya que las especies de bosques y matorrales presentaban un mayor riesgo térmico. A partir de nuestros resultados, hemos diseñado un marco para apoyar los esfuerzos de conservación y restauración que incluye medidas para mejorar la disponibilidad de datos (por ejemplo, análisis de lagunas de datos) y para clasificar las especies según su vulnerabilidad al calentamiento climático. Estas medidas ayudarán a distinguir entre las especies potencialmente adecuadas para programas de restauración y aquellas que deben ser prioritarias para la conservación, y demostrarán cómo la información sobre la germinación puede modelarse y traducirse en evaluaciones del riesgo térmico para apoyar los programas de conservación y restauración basados en semillas.

## 建立种子萌发数据模型以满足地中海地区的生物多样性保护需求

**【摘要】** 以种子为基础的保护和恢复计划依赖于对种子基础生物学的深入了解。然而, 尚未有研究充分探讨当前的种子萌发数据能否满足生物多样性保护需求。本研究评估了1495种具有保护和恢复价值的地中海植物的种子萌发数据可用性和质量, 以确定当前可获得的萌发数据信息、现有数据是否满足种子萌发热风险评估的要求, 以及哪些物种或生态特征有助于解释当前的数据空缺。为此, 我们采用了系统发育逻辑回归分析, 并通过对物种的种子萌发热响应进行建模, 以评估现有数据的充分性。我们发现, 31%的物种拥有种子萌发数据, 但仅有9%的物种数据足以进行热响应建模。这些物种基本都具有广泛的生态分布, 可产生生理休眠的种子, 其中包括适用于生态恢复的物种, 以及具有传统用途的物种。物种的热风险并未表现出系统发育聚类信号, 但与栖息地相关, 来自森林和灌木丛的物种表现出更高的热风险。基于以上研究结果, 我们构建了一个支持保护与恢复工作的框架, 包括提高数据可用性(如数据空缺分析)的步骤, 以及将物种按照其气候变暖脆弱性进行分类的步骤。这些步骤将有助于区分哪些物种可能潜在适用于恢复计划, 哪些物种应得到优先保护, 并展示了如何将萌发信息建模并转化为热风险评估, 以支持以种子为基础的保护与恢复计划。**【翻译:胡怡思;审校:聂永刚】**

## INTRODUCTION

Seeds play an important role in plant adaptation to changing environments, contributing to the ecosystems’ integrity and resilience (Aurelle et al., 2022). They help plants reach suitable habitats through dispersal (Muñoz‐Gallego et al., 2023), survive unfavorable periods via soil seed banks (Gioria et al., 2020), and germinate when environmental conditions are favorable for seedling survival (Baskin & Baskin, 2014). Thus, studying plant regeneration from seeds is crucial for understanding species reproduction under current and future climate scenarios (Baskin & Baskin, 2022; Walck et al., 2011) and provides critical information for biodiversity conservation (Price et al., 2024; White et al., 2025) and ecological restoration (Dalziell et al., 2022; Ladouceur et al., 2018; Pedrini & Dixon, 2020).

Germination occurs as a response to a combination of environmental cues (Baskin & Baskin, 2014), such as temperature, moisture, light, and seasonal signals, which together constitute the seed germination niche (Donohue et al., 2010; Grubb, 1977). Within this niche, temperature plays a major role in controlling the timing and success of germination (Baskin & Baskin, 2014). The thermal germination niche, defined by the optimum temperature and the range of temperatures under which germination occurs, reflects a combination of fixed adaptive traits and plastic responses that allow seeds to cope with varying environmental conditions (Donohue et al., 2010). Thus, modeling this niche allows quantification and comparison of germination responses to temperature variation across species and populations under a warming climate (Cochrane et al., 2011).

Germination niche modeling requires adequate and robust data (Kildisheva et al., 2020; Price et al., 2024), particularly to increase species representativeness across habitats (Cruz‐Tejada, Mattana, et al., 2024). Increasing amounts of seed germination data are being archived (Cruz‐Tejada, Fernández‐Pascual, et al., 2024; Fernández‐Pascual et al., 2023; Ordóñez‐Parra et al., 2022). These data support broadscale syntheses of the germination niche across multiple species, especially for temperate biomes (Carta et al., 2022; Fernández‐Pascual et al., 2021), such as the Mediterranean (Cruz‐Tejada, Mattana, et al., 2024). However, it is not clear whether these data are also adequate for species‐level niche modeling because the outcomes of such modeling approaches are highly influenced by the total number of germination tests (data availability) and the quality of the data (e.g., number of germination tests across a thermal gradient). Moreover, the availability and quality of seed germination data are usually not evenly distributed among species. Widely distributed species occurring across multiple habitat types are more likely to be studied because their broad spatial distribution or environmental tolerance makes them more accessible for seed sampling and therefore better represented in open‐access repositories. For species of conservation interest or those relevant for restoration, seeds are also expected to be more likely studied due to conservation and restoration priorities (European Commission, 1992). As a result, much of the available germination information for these species is generated through ex situ collections and experiments conducted in conservation seed banks (e.g., Mattana et al., 2023), which represent a major source of empirical knowledge on species’ regeneration requirements that support conservation planning and comparative germination studies (Carta et al., 2022). Incorporating species‐specific germination requirements in decision‐making frameworks can substantially improve the success of conservation translocations (Godefroid et al., 2025) and seed‐based ecological restoration actions (Pedrini & Dixon, 2020).

Gaps in germination data have direct conservation implications (Bucharova et al., 2025). For species with complex reproductive cycles, including pollination and dispersal strategies and deep dormancy, seed collection can be challenging, and subsequent processing and testing may be limited (Fenner & Thompson, 2005; Joffre et al., 2007). In this context, it is also important to assess whether variation in data availability and quality is phylogenetically structured and thus reflective of shared evolutionary history rather than ecological differences because species are not evolutionarily independent (Garamszegi, 2014). Although previous studies have explored broadscale germination responses or thermal risk patterns in temperate floras (Carta et al., 2022; Fernández‐Pascual et al., 2021) and tropical regions (Sentinella et al., 2020), no one has yet examined the availability, quality, and species‐level adequacy for modeling of germination data for a large Mediterranean species pool.

The Mediterranean basin is a biodiversity hotspot due to its unique geographical and environmental characteristics (Myers et al., 2000). The same characteristics make it particularly vulnerable to rising temperatures (Giorgi & Lionello, 2008), and with summer temperatures in the northern Mediterranean increasing at up to twice the rate of global warming (RCP8.5 is projected to be ∼4°C by 2100) (IPCC, 2022), Mediterranean ecosystems are considered to be highly sensitive to climate change (Thompson et al., 2026). In this context, evidence from comparable climate‐sensitive floras shows that assessing the thermal germination niche (Hirst et al., 2025) and, when relevant, integrating additional criteria, such as genetic risk, provide practical foundations for identifying species’ vulnerability and guiding ecological restoration decisions (Mattana et al., 2026). In Mediterranean ecosystems, warming‐driven shifts in germination phenology may directly reduce recruitment opportunities, thereby challenging seed‐based conservation and restoration actions. This underscores that biodiversity conservation requires a deep understanding of species‐specific seed biology (Just et al., 2024), including their germination requirements and sensitivity to temperature. Inadequate integration of species’ reproductive and germination constraints can compromise conservation translocation success across Europe (Godefroid et al., 2025). Recent evidence also highlights that conserving Mediterranean biodiversity requires safeguarding the evolutionary history through phylogenetically informed, diverse, and better‐coordinated conservation strategies (Carta et al., 2025; Thompson et al., 2026).

Our goal was to assess the availability and adequacy of germination data for thermal modeling of characteristic species in the Mediterranean, representing an essential species pool for the design of effective policy and practices for habitat restoration and biodiversity conservation. By using phylogenetic logistic models, we tested the following hypotheses: seed germination data are limited in quantity and quality for species exhibiting particular vegetative (life form), reproductive (fruit type, pollination), and seed (mass and dormancy) traits, hampering seed collection, processing, and germination; data quantity and quality are not a constraint for species of wide ecological breadth or for species of conservation interest or those commercially available for restoration. For those species with adequate data for thermal modeling, we estimated the risk to seed germination of increasing temperatures (hereafter *thermal risk*) with the aim of improving understanding of species responses to future global warming scenarios and conservation management outcomes. Based on our results, we identified key challenges related to the limited availability and quality of germination data and outlined potential solutions to address these limitations. We also identified a framework to support seed‐based conservation and restoration programs in the Mediterranean.

## METHODS

### Species pool and germination data

We used the 1495 characteristic species of Mediterranean habitats from the EUNIS expert system (Chytrý et al., 2020) as the potential pool of species of conservation and restoration interest in the Mediterranean. This list of species was originally compiled in Cruz‐Tejada, Fernández‐Pascual, et al. (2024) and subsequently reused and taxonomically standardized in Cruz‐Tejada, Mattana, et al. (2024), where data on seed germination for species were also compiled. No species were excluded. Thus, no additional matching, duplicate filtering, or taxonomic reconciliation was required. All species names follow the *World Checklist of Vascular Plants* (Govaerts et al., 2021).

### Data availability and quality predictors

We selected nine plant features as predictors of germination data availability and quality (Table 1). We focused on traits that are directly relevant to seed sampling, handling, curation, and germination experiments in conservation and restoration contexts. We gathered vegetative (i.e., life form), reproductive (i.e., fruit type and pollination), and seed (i.e., mass and dormancy) traits and species‐level information. This information, extracted from publicly available datasets and publications, included ecological breadth (i.e., number of habitats in which each species occurs) based on the EUNIS expert system (Chytrý et al., 2020); inclusion in European conservation policies (Habitats Directive); relevance for ecological restoration (i.e., commercially available for restoration [Ladouceur et al., 2018]); and reported plant uses (Table 1). The structure and coding of all predictors and their sources are provided in Table 1. Ecological breadth was calculated based on the number of habitats in which the species is present (i.e., three‐level hierarchical classification of European habitats [Chytrý et al., 2020]). When it was not possible to find information on reproductive and seed traits for a species, we used data from congeneric species. Species with missing trait information were assigned no information category when applicable. All predictors were analyzed in separate univariable phylogenetic logistic regression models. Traits that were conceptually categorical were encoded as binary dummy variables (0/1) and standardized prior to analysis. No multilevel categorical factors were fitted. Continuous predictors were analyzed as continuous variables and log transformed. The phylogenetic tree of the species pool and the trait distribution across lineages are in Appendix S1.

**TABLE 1:** Predictors of seed germination data availability (presence or absence).

Feature	Predictor	Predictor type (unit)	Hypothesis	Source of predictor information
Vegetative	Life form annual perennial herbaceous woody	Binary (1, 0)	Data availability for perennial herbs and annual plants is higher than for woody plants because perennials are easily found in natural areas and woody plants have complex reproductive cycles, often with either very large or fleshy dispersal units (Joffre et al., 2007).	www.FloraVeg.eu Govaerts et al., 2021
Reproductive	Pollination biotic abiotic	Binary (1, 0)	Species with biotic pollination (i.e., animal‐mediated pollination) are less studied and have very little germination data due to low seed production (e.g., influenced by pollinator availability and specificity; compatibility and competition between individuals) (Ashman et al., 2004).	Kattge et al., 2020
	Fruit type dehiscent indehiscent fleshy	Binary (1, 0)	Fleshy fruits often have complex morphological structures that involve additional treatments to allow germination, making them difficult to study in comparison with species with dehiscent and indehiscent fruits (Spjut, 1994).	Carta et al., 2024
Seed	Seed mass	Continuous (mg)	There is lower production of large seeds relative to small seeds; thus, smaller seeds are more likely to be studied because they are more available (Fenner & Thompson, 2005).	Carta et al., 2024
	Dormancy PY(PD) PD M(P)D ND NoInfo	Binary (1, 0)	Germination data on species with deep and complex seed dormancy are not available due to the difficulty of experimentally breaking dormancy (Baskin & Baskin, 2014).	Rosbakh et al., 2023
Ecological breadth	Number of habitats**	Continuous (n)	Species restricted to one or few habitats are less studied because they are more difficult to sample.	Chytrý et al., 2020
Conservation interest	Species of conservation interest	Binary (1, 0)	Species listed in the annexes of the Habitat Directive are more studied than others because they are of conservation concern.	European Commission, 1992
Restoration relevance	Species relevant for ecological restoration	Binary (1, 0)	Species relevant for ecological restoration are more studied than others.	Classification derived from the European restoration species pool proposed by Ladouceur et al. (2018)
Plant uses	Species with traditional uses	Binary (1, 0)	Species with traditional uses are more studied than others (Pironon et al., 2024).	Pironon et al., 2024

Abbreviations: PY(PD), physical and physical‐physiological dormancy (combined); PD, physiological dormancy; M(P)D, morphological and morphophysiological dormancy (combined); ND, nondormant; NoInfo, no information on seed dormancy reported. **The ecological breadth was calculated based on the number of habitats on which the species is present (i.e., three‐level hierarchical classification of European habitats [Chytrý et al., 2020])

We evaluated data availability across all species as a binary response (i.e., presence or absence of data for each species) with phylogenetic logistic regression models implemented in the r package phylolm (logistic MPLE) (Tung Ho & Ané, 2014). We fitted each predictor in a separate univariable model and extracted the alpha (α) parameter, which represents the strength of phylogenetic signal (i.e., the higher the value of α, the weaker the phylogenetic dependence). Then, we used multiple factor analysis (MFA) in the package FactoMineR (Le et al., 2009) to construct a multivariate ordination to visualize the cross‐covariance among our predictors of data availability for each species.

Using the function glmmTMB in the R package glmmTMB, we evaluated the adequacy of data for quantifying thermal germination responses at the species level with binomial mixed quadratic models (Sentinella et al., 2020) in which seed lot was a random factor (Brooks et al., 2017). To account for overdispersion, models included an observation‐level random effect and were fitted using a beta‐binomial error distribution. Zero inflation was not modeled because residual diagnostics did not indicate systematic excess zeros. Based on the outcome of these models, we were able to evaluate each species according to the thermal modeling criteria in Table 2. These criteria were based on the quadratic model parameters that reflected whether the species’ germination response followed the expected unimodal pattern with downward concavity. Uncertainty associated with derived thermal parameters (e.g., optimal temperature [*T*_opt_] and thermal breadth [*T*_breadth_]) was quantified using Sentinella et al.’s (2020) approach based on the delta method (see below). When derived thermal parameters or their uncertainty were not estimable (e.g., due to lack of a real solution at the selected germination threshold), values were denoted not applicable (NA). We then modeled data quality at the species level as a binary response (passed or failed quality assessment for each species)—that is, the data were either adequate or did not fit a reliable quadratic model (details in Table 2)—by fitting phylogenetic logistic models (logistic MPLE) (Tung Ho & Ané, 2014) in the same way we tested data quantity. Finally, we included the thermal modeling outcomes as supplementary variables in the MFA ordination to visualize the correspondence between availability and quality.

**TABLE 2:** Criteria used for thermal modeling to assess seed germination data quality.

Criterion	Description	Decision rule
Number of tested temperatures NTestTemp >2	Number of different germination temperatures tested for each species; if species has been tested at more than two different temperatures, NTestTemp is assigned a value 1; if species has been tested at two or fewer temperatures, NTestTemp is assigned a value 0	Species with >2 germination temperatures tested have high‐quality data (i.e., quality = 1)
Model concavity *a* < 0	*a* is the leading coefficient that determines the concavity of the quadratic curve; *a* > 0 indicates upward concavity, precludingthe identification of an optimum	Species with *a* > 0 do not fit models (i.e., quality = 0)
Tail drop (not flat) TestTemp > *T*_opt_ & propgerm <0.75*Pmax TestTemp < *T*_opt_ & propgerm <0.75*Pmax	Curves adequately shaped (not flat) when germination is at least 25% below the maximum on the low‐ and high‐temperature sides of *T*_opt_	Pass (quality = 1): quadratic response shows downward concavity with decline at both thermal extremes; fail (quality = 0): curve is flat or lacks tail decline

Abbreviations: TestTemp, germination temperatures tested; *T*_opt_, optimal temperature (temperature where the model predicts maximum germination); propgerm, proportion of germinated seeds; Pmax, maximum germination percentage for each species.

Although the phylogenetic regressions returned an estimate of phylogenetic signal for the response to given predictors, it was more practical to estimate the phylogenetic signal of both data availability and data quality if the signal was treated as binary response without inclusion of any predictor. We used the phylo‐D function (R package caper [Orme et al., 2023]) for this estimate.

### Thermal risks

For those species passing the quality assessment (i.e., data were adequate to fit a reliable quadratic model), we estimated thermal parameters, such as the *T*_opt_ and *T*_breadth_, with quadratic thermal modeling for each species (Sentinella et al., 2020). Uncertainty estimates for *T*_opt_ and *T*_breadth_ derived using the delta method were associated with species‐level thermal metrics and are reported as standard error (SE). We excluded species for which *T*_opt_ or *T*_breadth_ was not estimable, for which their associated SE could not be computed, or for which *T*_opt_ fell outside the experimentally tested temperature range from the thermal risk classification. We treated *T*_opt_ and *T*_breadth_ as point estimates derived directly from the fitted quadratic coefficients. Uncertainty estimates were used descriptively to assess model reliability and were not incorporated in the assignment of risk classes. Models yielding biologically unreliable optima were excluded through the three thermal quality criteria (Table 2), ensuring that only biologically meaningful estimates entered the risk assessment. Then, we calculated the mean of the optimal temperature (*T*_optM_) and of the optimal thermal breadth (*T*_breadthM_) for seed germination across all species: *T*_optM_ = 17°C and *T*_breadthM_ = 8°C. Using these two parameters as average thresholds across all species and accounting for their thermal limits, we allocated them to four risks categories: thermally resilient, species with a warm *T*_opt_ and wide *T*_breadth_ (*T*_opt_ > 17°C & *T*_breadth_ > 8°C); warm‐narrow risk, species with warm *T*_opt_ and narrow *T*_breadth_ (*T*_opt_ > 17°C & *T*_breadth_ ≤ 8°C); cold‐wide risk, species with cold *T*_opt_ and wide *T*_breadth_ (*T*_opt_ ≤ 17°C & *T*_breadth_ > 8°C); and high thermal risk, species with cold *T*_opt_ and narrow *T*_breadth_ (*T*_opt_ ≤ 17°C & *T*_breadth_ ≤ 8°C).

To evaluate whether these thermal risk categories were nonrandomly distributed across habitats, we tested the independence between the four risk classes and habitat types with chi‐square tests and Fisher's exact tests when expected frequencies were low.

We constructed a second MFA ordination to visualize the relationship between thermal limits (i.e., *T*_opt_, optimal low temperature, optimal upper temperature, and *T*_breadth_) and seed dormancy. Finally, we assessed the phylogenetic signal of thermal risk categories with the phylo‐D function and analyzed independence between thermal risk categories and habitat types separately with chi‐square (or Fisher's exact) tests, as described above.

## RESULTS

### Data availability and quality assessments

**FIGURE 1:**
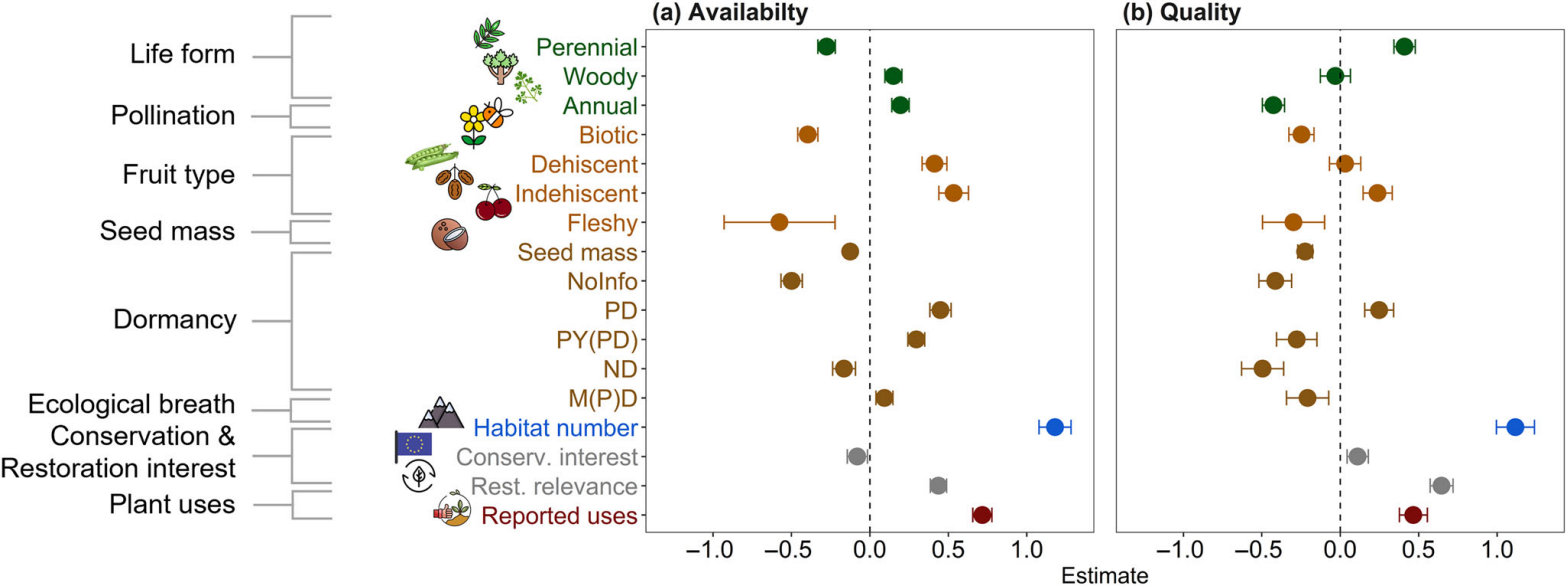
Estimated effect sizes of predictors related to germination data (a) availability (presence or absence) and (b) quality (based on criteria for thermal modeling) (predictor [left] groups: green, vegetative traits; orange, reproductive traits; brown, seed traits; blue, ecological breadth; gray, conservation concern and relevance for restoration; dark red, plant uses; whiskers are standard errors, SE); NoInfo, no information on seed dormancy reported; PY(PD), physical and physical‐physiological dormancy (combined); ND, nondormant; M(P)D, morphological and morfophysiological dormancy (combined). Each predictor was tested in an independent model.

Data were available for 31% of the 1495 species. Among these, 91% did not meet the criteria for data quality, leaving only 9% of species (140) with adequate data for modeling the thermal germination niche. Overall, most of our hypotheses concerning data availability and quality were supported (Figure 1). Seed germination data availability was limited for perennial species exhibiting reproductive (biotic pollination, fleshy fruits) and seed (seed mass, nondormant seeds) traits that hamper seed collection, processing, and germination. Species with large seeds were underrepresented, and, contrary to our expectations, species of conservation concern had limited data availability. Conversely, data were more abundant and adequate for thermal modeling for species with wide ecological breadths that set indehiscent fruits and had physiologically dormant (PD) seeds and for species relevant for restoration and that had traditional uses (Figure 1; Appendix S2). The multivariate ordination broadly agreed with these univariate regressions but offered the opportunity to summarize the cross‐covariance among variables (details in Appendix S3).

Data availability and quality showed a moderately significant phylogenetic structure (*D* = 0.765 and *D* = 0.898, respectively [*p* < 0.001]) (i.e., closely related species shared similar levels of data representation). Logistic models that included predictors with strong effects on data availability (e.g., perennial life form, biotic pollination, PD, ecological breadth, and plant uses) and data quality (e.g., seed mass, fruit type, dormancy, and ecological breadth) indicated a phylogenetically structured availability in germination data (α < 0.2) (Appendices S4 and S5). The full dataset of the 1495 species including trait data and quality assessment results is in Appendix S6.

### Thermal risks to seed germination

**FIGURE 2:**
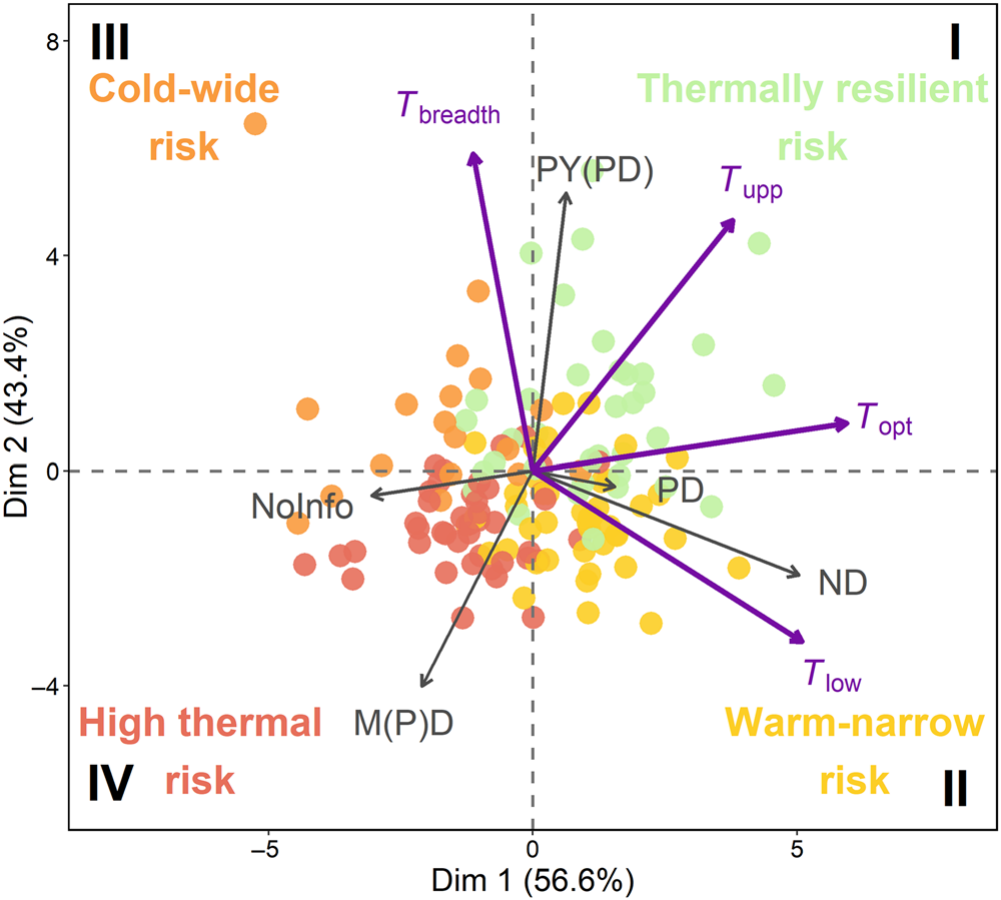
Results of a multiple factor analysis ordination summarizing the cross‐covariance among thermal parameters (i.e., optimal low temperature [*T*_low_], optimal temperature [*T*_opt_], and optimal upper temperature [*T*_upp_]); optimal thermal breadth (*T*_breadth_); and seed dormancy of plant species in the Mediterranean to visualize the potential thermal risks to seed germination (purple arrows, thermal parameters used to construct the ordination; black arrows, dormancy supplementary variables; I, thermally resilient species [36 species] not at risk; II, species [42] with limited thermal breadth but warm optimal temperatures; III, species [20] with a broad thermal breadth but low optimal temperatures; IV, species [42] with narrow thermal breadth and cold optimal temperatures; NoInfo, no information on seed dormancy reported; PY[PD], physical and physical‐physiological dormancy [combined]; ND, nondormant; M[P]D, morphological and morphophysiological dormancy [combined]). Species thermal risk categories are in Appendix S7.

The species ordination based on thermal parameters for the 140 species with adequate data showed that these species were unevenly distributed in the multidimensional space. Among the four thermal risk categories, 9% were thermally resilient, 25% were warm‐narrow, 31% were cold‐wide, and 35% were high risk (Figure 2; Appendix S7). The first ordination axis separated cooler *T*_opt_ species (Figure 2, left) from warmer *T*_opt_ species (Figure 2, right), which generally corresponded to those with PD and nondormant seeds. The second MFA axis separated narrow *T*_breadth_ species (i.e., species with morphological and morphophysiological dormancy: M[P]D) (Figure 2, bottom) from wide *T*_breadth_ species (Figure 2, top) (i.e., species with physical and physical‐physiological dormancy [combined]: PY[PD]).

The species within the four risk categories were randomly distributed across taxonomic families (Fisher's exact test with simulated *p*, *p* > 0.05) (Appendix S8) but not across habitat types (Appendix S9). Thermal risk categories did not exhibit significant phylogenetic signal (*D* = 0.93, 0.80, 0.75, and 1.1 for high thermal risk, cold‐wide risk, warm‐narrow risk, and thermally resilient species, respectively, *p* > 0.05), indicating that species with similar levels of risk are not more phylogenetically related than expected by chance. Significant deviations from independence between risk levels and habitat type were found for coastal (χ^2^ = 17.57, *p* < 0.05), Forests (χ^2^ = 20.07, *p* < 0.05), grasslands (χ^2^ = 13.93, *p* < 0.05), and scrublands (χ^2^ = 15.14, *p* < 0.05). Specifically, warm‐narrow species were overrepresented in wetlands and coastal habitats, whereas cold‐wide species were consistently underrepresented, particularly in coastal, forests, and scrublands. In contrast, species at high risk were more frequent than expected by chance in forests and scrublands, whereas thermally resilient species showed a relatively homogeneous distribution across habitats.

## DISCUSSION

Overall, data were more abundant and adequate for thermal modeling in ecologically widely distributed species setting PD seeds, for species relevant for restoration, and for species with reported traditional uses. Our finding that widespread common species with economic interest were more studied stands in contrast to a number of investigations in the Mediterranean that targeted threatened and endemic species are most common (Kadis et al., 2010; Luna et al., 2012; Picciau et al., 2019; Thanos & Doussi, 1995). This apparent discrepancy is likely driven by differences in study scope and sampling strategies; we targeted species characteristic of the Mediterranean rather than narrowly distributed taxa. By focusing on these species, our results are generalizable across the region rather than limited to a specific geographic area, and our methods provide a transferable framework for improving germination data to support seed‐based conservation and restoration programs.

### Germination data availability and quality

In line with our expectations, data were available and adequate for thermal modeling in ecologically widely distributed species because their broader distribution across diverse habitats likely facilitates their study due to increased accessibility. Likewise, it was not surprising that species whose seeds were relevant for restoration exhibited high data availability and were adequate for thermal modeling because understanding their germination requirements is crucial for achieving successful restoration efforts (Dalziell et al., 2022; Hirst et al., 2025; Pedrini & Dixon, 2020). However, the sustainability of harvesting seeds from wild populations to supply this demand is increasingly questioned, and guidelines based on species’ generation time have been proposed to mitigate demographic risks (Bucharova et al., 2025).

Several consistent patterns of data scarcity emerged across species. Data availability was low for perennial herbs, particularly those with biotic pollination. Their reliance on a limited number of pollinators for reproduction success may constrain seed production relative to abiotic pollination (i.e., unassisted and self‐pollination), thereby reducing opportunities for germination testing. Pollinator‐dependent species are disproportionately vulnerable to environmental change and population decline, leading to reduced seed production and recruitment, which is consistent with their underrepresentation in experimental and conservation datasets (Ashman et al., 2004; Eckert et al., 2009). Data on germination in species setting fleshy fruit were limited (consistent with our hypothesis), likely because their complex morphological and functional features limit their seed processing and germination (Baskin & Baskin, 2014) (Table 1). Plants with large seeds were also underrepresented, likely due to their low seed production or the large space needed to conduct experiments (Fenner & Thompson, 2005). Species of conservation concern had similarly limited data, probably due to difficulties in seed collection and legal restrictions, which may hinder their inclusion in conservation planning. Surprisingly, species with nondormant seeds also showed limited data availability, despite their lack of specific germination requirements. Thus, their lower ecophysiological complexity may lead researchers to prioritize dormant species (Shefferson & Tali, 2007). Because many conservation and restoration practices rely on managing dormancy to enhance seed germination success (Hirst et al., 2025; Kettenring & Tarsa, 2020; Kildisheva et al., 2020), this likely explains why species with dormant seeds were more studied (White et al., 2025).

Broadly, species selection for conservation and restoration programs often relied on habitat rarity, population trends, feasibility of seed collection, or expert knowledge, rather than germination knowledge alone (Diallo et al., 2021; Fenu et al., 2023; Pérez‐Navarro et al., 2023). Nevertheless, species whose regeneration requirements are poorly known were frequently excluded from conservation and restoration initiatives (Kildisheva et al., 2020). In line with regional evidence (Fenu et al., 2023; Pérez‐Navarro et al., 2023; Thompson et al., 2026), our results showed that germination data gaps were not randomly distributed; rather, they reflected consistent ecological and practical constraints and were concentrated in specific trait‐based groups, particularly perennial herbs, fleshy‐fruited species, and biotically pollinated taxa.

### Trade‐off between data availability and quality

Overall, when germination data were available, they were also of adequate quality for thermal modeling, but this was not always the case. For example, although germination data were scarce for most perennial herbs and conservation species, when available for a given species, they were adequate for thermal modeling. As such, available data for perennial conservation species are of high research value for informing management strategies, despite sampling challenges linked to their small population sizes and permit restrictions (Bartholomew et al., 2023; Heywood & Iriondo, 2003). Conversely, annuals and species setting dehiscent fruits and PY(PD) exhibited high data availability but low data quality, limiting their adequacy for thermal modeling. These contrasting patterns highlight that species’ reproductive traits critically shape, in opposite ways, the quantity and quality of germination data to inform conservation programs.

### Thermal risks to seed germination

Although only 140 species passed the quality assessment, these species provided a valuable dataset for modeling thermal limits and exploring thermal risks. These thermal risk categories represented relative, rather than absolute, vulnerability. Nevertheless, modeling thermal responses at the species level remains essential because it captures biologically meaningful variation among species (and among populations when data are available), providing a comparative framework for assessing sensitivity to warming in relation to species’ habitat context and climatic setting (Hirst et al., 2025; Thompson et al., 2026). Species potentially at high thermal risk (i.e., exhibiting a cold and narrow germination niche) often lacked dormancy information or exhibited complex dormancy types, such as M(P)D, highlighting priority targets for future germination research.

The majority of species relevant for restoration did not exhibit high thermal risk, indicating that these taxa are likely to remain functional under projected warming and can therefore be considered resilient candidates for restoration. Likewise, none of the species currently identified as of conservation interest were classified as high thermal risk, partly reflecting data limitations that still preclude germination risk assessments for many conservation priority taxa. These findings demonstrated that thermal risk could identify additional taxa that warrant conservation attention beyond existing conservation frameworks (Hirst et al., 2025) and call for stronger collaboration across stakeholders to enhance seed supply systems and ensure access to diverse, high‐quality seeds for conservation and restoration (Andres et al., 2024).

Our results suggest that risk categories are mainly influenced by habitat rather than phylogeny, meaning more attention to critical ecosystems is needed. Indeed, species at high thermal risk were most frequent in forests and scrublands, highlighting their urgent need for conservation. In contrast, warm‐narrow species were overrepresented in coastal and wetland habitats, reflecting restricted thermal tolerances. Although these species currently face a lower thermal risk, their persistence could be threatened if temperatures shift beyond their tolerance limits. Their prevalence makes them valuable candidates for future restoration programs, yet targeted conservation and monitoring in coastal areas remain essential to ensure their long‐term stability under climate change.

### Challenges and perspectives

**FIGURE 3:**
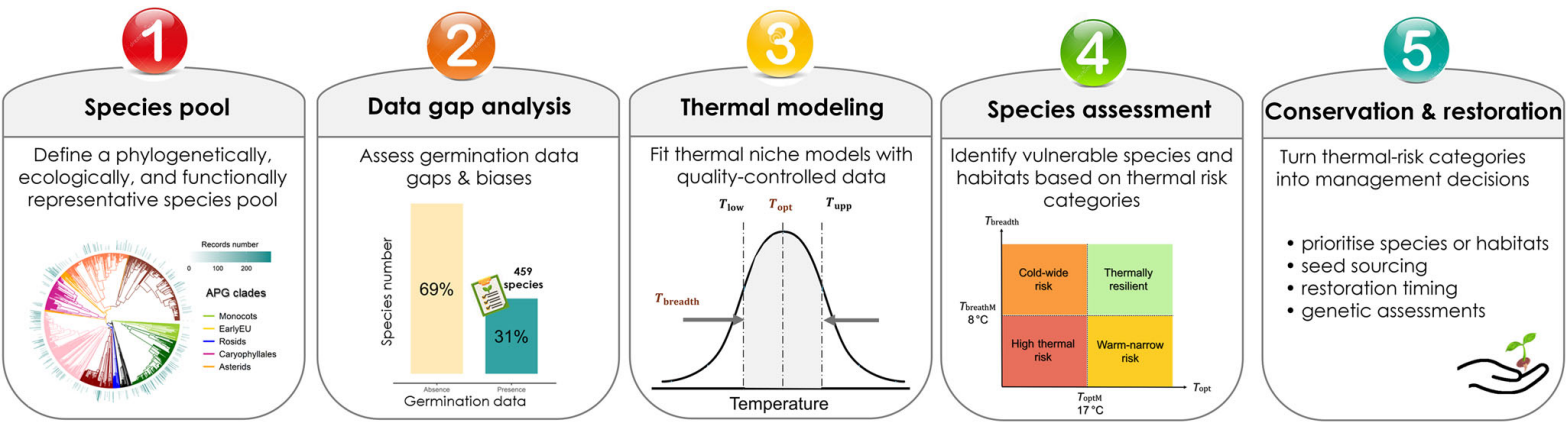
The five steps for strengthening germination evidence to support conservation and restoration in the Mediterranean basin, linking germination ecology to applied biodiversity conservation (optimal low temperature [*T*_low_]; optimal temperature [*T*_opt_]; upper temperature [*T*_upp_]; mean optimal temperature [*T*_optM_] ; mean thermal breadth [*T*_breadthM_: range between lower and upper thermal limits]; thermally resilient species, species with seed germination not at risk from increasing temperatures; warm‐narrow risk, species with limited thermal breadth but warm optimal temperatures; cold‐wide risk, species with a broad thermal breadth but low optimal temperatures; high thermal risk, species with narrow thermal breadth and cold optimal temperatures).

Our findings showed that limitations in germination data availability and modeling adequacy are structured across traits and habitats, currently constraining the effective use of seed‐based information in conservation and restoration planning. To address these limitations, we propose a framework (Figure 3) that integrates data generation, thermal germination modeling, and decision‐making. This framework links data gaps, thermal germination modeling, and species‐level risk assessment with outputs translated into actionable guidance for conservation and restoration, including species and habitat prioritization, climate‐informed seed sourcing, and, where possible, the integration of complementary genetic information to refine management decisions (Mattana et al., 2026). By embedding thermal germination modeling within a broader assessment of species vulnerability and habitat context, this approach supports the identification of priority taxa and habitats, guides targeted data collection efforts, and facilitates the incorporation of thermal germination risk assessments into restoration and conservation strategies. More broadly, this framework provides a transferable approach for strengthening the role of seed biology in seed‐based biodiversity conservation under ongoing climate change and highlights the urgent need to generate robust germination data for underrepresented groups and to improve experimental and modeling quality where data availability alone is the limiting factor (Godefroid et al., 2025; Hirst et al., 2025; Mattana et al., 2026; Thompson et al., 2026). We showed that germination information can be modeled and translated into thermal risk assessments to support seed‐based conservation and restoration programs. A strategic shift is needed to focus research on neglected taxa, improve data integration, and foster collaboration among scientists, practitioners, and policy makers in the Mediterranean and in other climate‐sensitive regions worldwide.

## Supporting information




**Supplementary Materials**.

**Supplementary Materials**.

**Supplementary Materials**.

## Data Availability

The original germination data are in Cruz‐Tejada, Mattana, et al. (2024). The trait dataset generated for the analyses and the thermal modeling results are in the Supporting Information and are available in GitHub (https://github.com/DianaCruzTejada/MedGerm_DataAssessment), where the R codes used for the analyses are also available. A versioned archive of the repository is available in Zenodo (https://doi.org/10.5281/zenodo.20136467)
