# Supplementary material for: Modeling seed germination data to meet biodiversity conservation needs in the Mediterranean
Source: Conserv Biol. 2026 May 18;40(5):e70329. doi: 10.1111/cobi.70329 (PMC13613545; doi:10.1111/cobi.70329)
Supplement: Supplementary file 1 [file COBI-40-e70329-s002.docx]

### **SUPPLEMENTARY MATERIAL**

**Modeling seed germination data to meet biodiversity conservation needs in the Mediterranean**

**Appendix S1.** Phylogenetic distribution of the 1495 Mediterranean species with (a) information of vegetative, reproductive and seed traits, (b) conservation and restoration species information, and (c) plant use categories.

**Appendix S2.** Number and percentage of species exhibiting vegetative, reproductive and seed traits, conservation and restoration species information and plant use categories.

**Appendix S3.** Multiple Factor Analysis (MFA) summarizing the cross-covariance between predictors of data availability and thermal modeling criteria to assess data quality.

**Appendix S4.** Binary phylogenetic generalized linear models results testing the effect of our predictors on the presence or absence of germination. Each predictor (i.e., row) follows an independent model.

**Appendix S5.** Binary phylogenetic generalized linear models results testing the effect of our predictors on the quality of germination data. Each predictor (i.e., row) follows an independent model.

**Appendix S6.** Full dataset of the 1,495 species analyzed, including trait data and quality assessment results (.csv file).

**Appendix S7.** Risk categories for 140 Mediterranean species. The table highlights species of conservation interest (i.e., listed under the Habitats Directive 92/43/EEC; European Commission, 1992) and species whose seeds are commercially available for ecological restoration (.csv file).

**Appendix S8.** Phylogenetic distribution of risk category of 139 Mediterranean species.

**Appendix S9.** Proportion of species across risk in the Mediterranean habitats. The color intensity represents the proportion of species in each risk category for each habitat, with darker shades indicating higher proportions.

**Appendix S1.** Phylogenetic distribution of the 1495 Mediterranean species with information on (a) vegetative, reproductive and seed traits, (b) conservation and restoration information, and (c) plant use categories (Diazgranados et al., 2020). The phylogenetic tree represents a wide phylogenetic dispersion, including the most representative clades of the angiosperms and it was generated based on the reference megatree (Smith & Brown, 2018) and plotted with the ggtree R package (Yu et al., 2017) with information on main clades as in the Angiosperms Phylogeny Group (APG IV, 2016)*.*

**Appendix S2.** Number and percentage of species exhibiting vegetative (green), reproductive (orange) and seed traits (brown), conservation (blue) and restoration (gray) information and plant use categories (red) (Diazgranados et al., 2020). The proportion of species with data availability is shown in turquoise, and the species without data are represented in gray (as indicated).

**Appendix S3.** Multiple Factor Analysis (MFA) summarizing the cross-covariance between predictors of data availability and thermal modeling criteria to assess data quality. Predictors are grouped into *features*, which are indicated at the bottom of the figure. (a, b) Predictors of data availability used to construct the ordination. (c) Thermal modeling criteria to assess data quality as supplementary variables not used to construct the ordination, showing the correspondence between data availability and quality. Dots represent species colored by the presence or absence of data (as indicated). Arrows correspond to loadings of the predictors. Iconic plant use categories are shown in (b), as reported in Diazgranados et al. (2020). The first MFA axis (14.9% of variability explained) as aligned with, on the right, woody species producing heavier seeds with M(P)D, PY(PD), and on the left, annuals and species setting dehiscent fruits. The second axis (12.2% of variability explained) roughly separates species for which germination data are available (bottom: species producing indehiscent fruits with wide ecological breadth and PD seeds) from those lacking germination data (top: biotically pollinated species producing fleshy fruits). Although this separation is supported by traditional plant uses and data quality, all pointing to the lower part of the ordination, the low overall variability explained, restricts the visibility to a feature combination of data availability shown by three main corners where species are grouped. Most species with available data adequate to fit thermal modeling are located on the lower part of the ordination. PY(PD), physical and physical-physiological dormancy (combined); PD, physiological dormancy; M(P)D, morphological and morphophysiological dormancy (combined); NTestTemp, number of tested temperatures.

**Appendix S4.** Binary phylogenetic generalized linear models results testing the effect of our predictors on the presence or absence of germination. Each predictor (i.e., row) is analyzed in an independent model. Alpha (α) indicates the phylogenetic signal estimated using maximum penalized likelihood (MPLE); lower α values suggest stronger phylogenetic dependence of the trait, meaning that closely related species tend to show similar values for the predictor.

| **Predictor** | **Estimate** | **SE** | **z** | **P** | **Alpha(α)** | **Lower** | **Upper** |
| --- | --- | --- | --- | --- | --- | --- | --- |
| **Life form** |  |  |  |  |  |  |  |
| Perennial | -0.276 | 0.056 | -4.970 | **<0.001** | 0.185 | -0.332 | -0.220 |
| Woody | 0.15 | 0.054 | 2.797 | **<0.01** | 0.291 | 0.096 | 0.204 |
| Annual | 0.195 | 0.055 | 3.565 | **<0.001** | 0.318 | 0.140 | 0.250 |
| **Pollination** |  |  |  |  |  |  |  |
| Biotic | -0.396 | 0.064 | -6.203 | **<0.001** | 0.160 | -0.460 | -0.332 |
| **Fruit type** |  |  |  |  |  |  |  |
| Dehiscent | 0.412 | 0.079 | 5.202 | **<0.001** | 0.085 | 0.333 | 0.491 |
| Indehiscent | 0.534 | 0.094 | 5.657 | **<0.001** | 0.049 | 0.440 | 0.628 |
| Fleshy | -0.576 | 0.354 | -1.63 | 0.103 | 0.005 | -0.93 | -0.222 |
| **Seed mass** |  |  |  |  |  |  |  |
| Seed mass | -0.125 | 0.032 | -3.917 | **<0.001** | 0.145 | -0.157 | -0.093 |
| **Dormancy** |  |  |  |  |  |  |  |
| NoInfo | -0.499 | 0.068 | -7.372 | **<0.001** | 0.196 | -0.567 | -0.431 |
| PD | 0.450 | 0.068 | 6.582 | **<0.001** | 0.102 | 0.382 | 0.518 |
| PY(PD) | 0.296 | 0.054 | 5.451 | **<0.001** | 0.390 | 0.242 | 0.350 |
| ND | -0.165 | 0.073 | -2.257 | **<0.01** | 0.286 | -0.238 | -0.092 |
| M(P)D | 0.093 | 0.054 | 1.741 | **<0.01** | 0.378 | 0.039 | 0.147 |
| **Ecological breadth** |  |  |  |  |  |  |  |
| Habitat number | 1.180 | 0.102 | 11.561 | **<0.001** | 0.119 | 1.078 | 1.282 |
| **Conservation and restoration interest** |  |  |  |  |  |  |  |
| Conservation interest | -0.080 | 0.064 | -1.248 | 0.212 | 0.390 | -0.144 | -0.016 |
| High-interest Restoration | 0.437 | 0.052 | 8.434 | **<0.001** | 0.142 | 0.385 | 0.489 |
| **Plant uses** |  |  |  |  |  |  |  |
| Reported uses | 0.717 | 0.061 | 11.670 | **<0.001** | 0.089 | 0.656 | 0.778 |

**Appendix S5.** Binary phylogenetic generalized linear models results testing the effect of our predictors on the quality of germination data. Each predictor (i.e., row) is analized in an independent model. Alpha (α) indicates the phylogenetic signal estimated using maximum penalized likelihood (MPLE); lower α values suggest stronger phylogenetic dependence of the trait, meaning that closely related species tend to show similar values for the predictor.

| **Predictor** | **Estimate** | **SE** | **z** | **P** | **Alpha(α)** | **Lower** | **Upper** |
| --- | --- | --- | --- | --- | --- | --- | --- |
| **Life form** |  |  |  |  |  |  |  |
| Perennial | 0.410 | 0.068 | 6.025 | **<0.001** | 0.016 | 0.342 | 0.478 |
| Woody | -0.031 | 0.096 | -0.326 | 0.745 | 0.399 | -0.127 | 0.065 |
| Annual | -0.426 | 0.071 | -6.012 | **<0.001** | 0.016 | -0.497 | -0.355 |
| **Pollination** |  |  |  |  |  |  |  |
| Biotic | -0.247 | 0.08 | -3.089 | **<0.01** | 0.399 | -0.327 | -0.167 |
| **Fruit type** |  |  |  |  |  |  |  |
| Dehiscent | 0.031 | 0.1 | 0.308 | 0.758 | 0.399 | -0.069 | 0.131 |
| Indehiscent | 0.238 | 0.093 | 2.571 | **<0.01** | 0.4 | 0.145 | 0.331 |
| Fleshy | -0.298 | 0.198 | -1.502 | 0.133 | 0.001 | -0.496 | -0.1 |
| **Seed mass** |  |  |  |  |  |  |  |
| Seed mass | -0.224 | 0.047 | -4.761 | **<0.001** | 0.402 | -0.271 | -0.177 |
| **Dormancy** |  |  |  |  |  |  |  |
| NoInfo | -0.414 | 0.104 | -3.989 | **<0.001** | 0.4 | -0.518 | -0.31 |
| PD | 0.248 | 0.092 | 2.681 | **<0.01** | 0.402 | 0.156 | 0.34 |
| PY(PD) | -0.277 | 0.129 | -2.143 | **<0.01** | 0.398 | -0.406 | -0.148 |
| ND | -0.495 | 0.134 | -3.699 | **<0.001** | 0.016 | -0.629 | -0.361 |
| M(P)D | -0.208 | 0.135 | -1.538 | 0.124 | 0.402 | -0.343 | -0.073 |
| **Ecological breadth** |  |  |  |  |  |  |  |
| Habitat number | 1.117 | 0.121 | 9.237 | **<0.001** | 0.388 | 0.996 | 1.238 |
| **Conservation and restoration interest** |  |  |  |  |  |  |  |
| Conservation interest | 0.111 | 0.068 | 1.632 | 0.103 | 0.401 | 0.043 | 0.179 |
| High-interest Restoration | 0.646 | 0.073 | 8.91 | **<0.001** | 0.395 | 0.573 | 0.719 |
| **Plant uses** |  |  |  |  |  |  |  |
| Reported uses | 0.466 | 0.089 | 5.215 | **<0.001** | 0.401 | 0.377 | 0.555 |

**Appendix S8.** Phylogenetic distribution of risk categories for 140 Mediterranean species. The tree was generated based on the reference megatree (Smith & Brown, 2018) and plotted with the ggtree R package (Yu et al., 2017) with information on main clades as in the Angiosperms Phylogeny Group (APG IV, 2016)*.* The species within the four risk categories are randomly distributed across taxonomic families (Fisher’s exact test with simulated p-value, *p* > 0.05. Thermal risk categories did not exhibit phylogenetic signal (D = 0.93; 0.80; 0.75; 1.1; p > 0.05, for high-thermal risk, cold-wide risk, warm-narrow risk and thermally resilient, respectively), indicating that species with similar levels of risk are not more phylogenetically related than expected by chance.

**Appendix S9.** Proportion of species across risk in the Mediterranean habitats. The color intensity represents the proportion of species in each risk category for each habitat, with darker shades indicating higher proportions. Proportions were calculated based on the species frequency in each category relative to the total number of species observed in each habitat. The Chi-squared test showed significant deviations from independence between risk and habitat type for Coastal (χ² = 17.57, p < 0.05), Forests (χ² = 20.07, p < 0.05), Grasslands (χ² = 13.93, p < 0.05), and Scrublands (χ² = 15.14, p < 0.05). No significant deviations were detected for Man-made (χ² = 0.73, p > 0.05) and Wetlands (χ² = 6.00, p > 0.05).

**References**

APG IV. (2016). An update of the Angiosperm Phylogeny Group classification for the orders and families of flowering plants: APG IV. *Botanical Journal of the Linnean Society*, *181*(1), 1–20. https://doi.org/10.1111/boj.12385

Diazgranados, M., Allkin, B., Black, N., Cámara-Leret, R., Canteiro, C., Carretero, J., Eastwood, R., Hargreaves, S., Hudson, A., Milliken, W., Nesbitt, M., Ondo, I., Patmore, K., Pironon, S., Turner, R., & Ulian, T. (2020). World checklist of useful plant species. In *Knowledge Network for Biocomplexity*.

Smith, S. A., & Brown, J. W. (2018). Constructing a broadly inclusive seed plant phylogeny. *American Journal of Botany*, *105*(3), 302–314. https://doi.org/10.1002/ajb2.1019

Tung Ho, L. S., & Ané, C. (2014). A linear-time algorithm for gaussian and non-gaussian trait evolution models. *Systematic Biology*, *63*(3), 397–408. https://doi.org/10.1093/sysbio/syu005

Yu, G., Smith, D. K., Zhu, H., Guan, Y., & Lam, T. T. Y. (2017). Ggtree: an R Package for Visualization and Annotation of Phylogenetic Trees With Their Covariates and Other Associated Data. *Methods in Ecology and Evolution*, *8*(1), 28–36. https://doi.org/10.1111/2041-210X.12628
